# DA-CycleGAN: Degradation-Adaptive Unpaired Super-Resolution for Historical Image Restoration

**DOI:** 10.3390/jimaging12040155

**Published:** 2026-04-03

**Authors:** Lujun Zhai, Yonghui Wang, Yu Zhou, Suxia Cui

**Affiliations:** 1 Department of Electrical and Computer Engineering, Prairie View A&M University, Prairie View, TX 77446, USA; 2 Department of Computer Science, Prairie View A&M University, Prairie View, TX 77446, USA

**Keywords:** historical image restoration, super-resolution, unpaired learning

## Abstract

Historical images as the dominant method for documenting the world and its inhabitants can help us to better understand the real history. Due to the limited camera technology, historical images captured in the early to mid-20th century tend to be very blurry, unclear, noisy, and obscure. The goal of this paper is to super-resolve images for historical image restoration. Compared to the degradations in modern digital imagery, those in historical images have unique features that are typically much more complex and less well understood. The discrepancy between historical images and modern high-definition digital images leads to a significant performance drop for existing super-resolution (SR) models trained on modern digital imagery. To tackle this problem, we propose a new method, namely DA-CycleGAN. Specifically, the DA-CycleGAN is built on top of CycleGAN to achieve unsupervised learning. We introduce a degradation-adaptive (DA) module with strong, flexible adaptation to learn various unknown degradations from samples. Moreover, we collect a large dataset containing 10,000 low-resolution images from real historical films. The dataset features various natural degradations. Our experimental results demonstrate the superior performance of DA-CycleGAN and the effectiveness of our image dataset for achieving accurate super-resolution enhancement of historical images.

## 1. Introduction

The study of historical images has attracted significant interest, as they played a vital role in documenting the world’s progress from the 1890s through the 1970s. However, unlike modern digital images, historical images were captured by low-quality devices due to immature camera technology and exhibited extraneous features, e.g., severe blurriness, noise, and haziness. Hence, it is of interest to super-resolve the historical images taken under such particular conditions.

In recent years, single-image super-resolution (SISR) algorithms have shown great success in the modern image SR task, owing to the powerful feature learning capability of deep neural networks [1,2,3,4]. SISR is a task that aims to reconstruct a higher-resolution (HR) image from a single low-resolution (LR) image, while refining textures and details to improve visual quality. As a typical ill-posed problem, SR is highly coupled with the degradation estimation. Most existing deep learning-based methods assumed that the degradation of LR is known and fixed (e.g., bicubic downsampling). Moreover, neural networks’ training heavily relies on synthetic data due to the lack of real-world degraded data corresponding to the HR image, leading the learning process to miss real degradation information. In spite of their success on benchmark datasets [5,6,7], the severe performance drop on historical LR images degraded by unknown processes limits their application. Therefore, historical image super-resolution (historical image SR), the restoration and resolution enhancement of archival images captured with early camera technologies, remains a challenging problem. Unlike modern digital images, historical images often contain complex and unknown degradations introduced by physical aging, film scanning, optical blur, chemical noise, and compression artifacts. These degradations significantly differ from commonly assumed synthetic degradations (e.g., bicubic downsampling), making it difficult for conventional SR models to generalize effectively.

SR algorithms can be divided into two different categories: blind SR and non-blind SR. Non-blind SR algorithms assume the degradation is known and reconstruct the HR image with the given blur kernel. To deal with multiple degradations, non-blind SR methods [8,9,10,11] rely on deep learning-based methods to directly learn a mapping from low- to high-resolution images. They were trained on a large set of predefined degradation data (e.g., combinations of noise, motion blurs, and Gaussian blurs within a fixed level range). These desirable non-blind SR results can be obtained only when the real degradation is known a priori.

In contrast, blind SR algorithms typically try to infer unknown degradation and super-resolve LR images. The accurate estimation of the downsampling kernel from internal or external similar patches is essential for the SR task [12,13]. Although recent blind SR methods have produced promising results for limited forms of degradation (e.g., blur), natural LR images in real applications are not always degraded to such an extent. When the degradation estimation differs from the true degradation, the estimation error can be further amplified by the blind SR process, producing undesired artifacts (e.g., over-sharpening and over-smoothing) [14]. To correct the estimated degradation error, several blind SR methods [12,14,15,16] were proposed. By iteratively correcting the degradation, artifact-free results can be gradually produced. The main drawback of these methods is that numerous iterations in the test phase are time-consuming. Unlike the above methods that focus on the degradation estimation from an LR image, we propose a different approach by employing a degradation adaptive module with strong and flexible adaptation to various degradations in real applications.

SR methods can be further categorized by network architecture: CNNs, GANs, and Transformers. Recently, various CNN-based SR methods have been well developed and have shown impressive results that surpass those of traditional SR methods [17]. SRCNN [18] is a pioneer CNN work in SR applications and demonstrated its vast superiority owing to CNN’s powerful feature learning capability. However, CNN-based SR methods [3,19,20,21] largely focus on minimizing the mean squared error (MSE), causing the reconstructed HR image to lack high-frequency details. To address this problem, SR using a generative adversarial network (SRGAN) [22] was proposed, and the perceptual loss function was introduced to produce HR images with finer texture details [23]. To address the absence of LR-HR image pairs problem, unpaired CycleGAN SR methods [24,25,26,27,28] were proposed. However, these CycleGANs have a synthetic-to-real generalization problem due to the model’s lack of robustness and stability across different test images. Transformer [29] was first developed for natural language processing (NLP) and was recently introduced into the SR field, and its variants have achieved impressive performance on benchmark datasets. However, transformer-based methods [30,31] rely on paired data to train the models, and the pre-trained models have severe performance degradation on historical image SR.

Existing SR methods have two major difficulties in the application of historical image SR. First, there is no effective SR model that has flexible adaptation to various unknown degradations in historical images. Second, training data with historical image degradations is not prepared. In most existing SR studies, synthetic pseudo-degradation is commonly used, in which low-resolution images are generated by artificially applying predefined operations, such as bicubic downsampling, Gaussian blur, and additive noise, to high-resolution images. Although convenient for supervised training, such artificially designed degradations cannot fully represent the complex and unknown degradation processes observed in real historical images [8,32]. Therefore, the absence of learning samples will directly lead to the failure of super-resolving historical images.

To overcome this, we first propose using the degradation-adaptive (DA) block, which provides powerful, flexible adaptation to complex historical image degradations. It can learn detailed features and then reconstruct HR images with finer details and textures. Additionally, to provide various historical image degradations for network training, we collected an LR image training dataset from a large set of historical films. The film cameras had existed for many decades before the current popular digital cameras. Due to the low quality of video capture devices, clarity, noise, and blurriness are exacerbated. Therefore, the degradations formed directly from the physical world have richer and more natural features, in contrast to the synthetic fixed degradations.

The main contributions of this work are summarized as follows:

- We propose a degradation-adaptive (DA) module that dynamically modulates convolutional kernels and channel responses to handle complex and unknown degradations in historical images.
- We design an unpaired super-resolution (SR) framework tailored for historical image restoration, avoiding reliance on synthetic degradation assumptions and improving robustness to real-world degradation.
- We construct a real historical low-resolution (LR) dataset collected from archival films, which better represents physically induced degradations compared to artificially generated pseudo degradations.
- Extensive experiments on historical images and benchmark datasets demonstrate that the proposed method achieves improved robustness and generalization compared with state-of-the-art unpaired SR approaches.

## 2. Related Work

In this section, we categorize existing SR methods into three main groups: (1) supervised SISR methods relying on paired LR–HR data, (2) unsupervised or unpaired SR approaches designed to overcome the lack of paired training data, and (3) SR methods addressing multiple or unknown degradations in real-world scenarios. We review these categories below and discuss their limitations in the context of historical image restoration.

### 2.1. Single Image Super-Resolution (SISR)

SISR, as a classical ill-posed inverse problem in computer vision, aims to reconstruct a high-resolution (HR) image from a single low-resolution (LR) image [33]. Learning-based methods have been the mainstream in SISR in recent years owing to the powerful feature extraction and representation learning ability of deep neural networks. The first work using the neural network method to solve the SISR problem can be traced back to when a three-layer SR CNN (SRCNN) was proposed. Since then, a category of CNN-based approaches has been extensively developed and has produced superior results. Kim et al. [19] presented a 20-layer network (VDSR), adapting a residual learning strategy. EDSR [34] further removed batch normalization (BN) layers to generate artifact-free SR results. To overcome the main drawback of CNN-based SR methods, which produce over-sharpened or over-smoothed SR results, SRGAN [22] introduced a perceptual loss to improve the perceptual quality of SR results. Residual-in-Residual Dense Block (RRDB) was introduced into ESRGAN [35] and further improved the quality of reconstructed images. More recently, transformer-based and diffusion-based methods have further advanced image super-resolution. For example, Swin2SR [36,37] explored the use of Swin Transformer V2 to improve super-resolution and restoration performance, especially for compressed inputs. In addition, diffusion-based super-resolution methods have shown strong potential in recovering finer details and improving perceptual quality by leveraging powerful generative priors. However, Real-world HR-LR image pairs are usually not available for preparing a training dataset. To address this problem, unpaired GAN-based SR methods [24,25,26,27] were proposed. Pseudo-CycleGAN [28] separated the GAN network into an unpaired noise correction CycleGAN and a pseudo-paired SR network to produce photo-realistic LR images well. However, due to the lack of flexibility to adapt to various complex degradations in historical images, the Pseudo-CycleGAN has a performance drop in historical image SR.

### 2.2. Unsupervised Image Super-Resolution

Most existing deep learning-based SR methods rely on LR-HR image pairs to train networks in a supervised manner. However, image pairs usually are absent in the physical world. Typically, to solve this problem, the bicubic downsampling method was widely employed in most studies to synthesize the corresponding LR image given an HR image. Recently, a few works [24,25,26,27,28] have proposed to adapt an unsupervised strategy to train SR networks. Inspired by the image-to-image translation application, Yuan et al. [24] proposed a Cycle-in-Cycle SR network (CinCGAN) that first learns a mapping from the noisy and blurry input to a noise-free LR space, and then upscales the clean LR to generate HR. Bulat et al. [25] adopt a two-stage strategy that first trains a High-to-Low GAN to degrade and downsample HR images in an unpaired manner and then uses the output of the High-to-Low GAN to train a Low-to-High GAN for SR. DNSR [26] proposed a degradation module to imitate the real-world degradation process from HR to LR via a GAN network. The generated photo-realistic LR images paired with real-world HR images are used as training data. To reduce the artifact caused by bicubic downsampling, Lugmayr et al. [27] proposed to invert the effects of bicubic downsampling and generate realistic image pairs for training using a GAN model. To generate more realistic images, [28] adopt a pseudo supervision strategy to solve the unpaired problem, and the noise correction network is mainly used to handle the LR image cleaning and then feed the generated clean LR image to the upscaler to generate the SR image.

Beyond SR, unsupervised and self-supervised learning strategies have also been explored in other challenging imaging scenarios involving complex degradations. For example, recent work on polarimetric binocular three-dimensional imaging in turbid water [38] employs a multi-feature self-supervised learning framework to recover structural information under severe scattering conditions. Although this work focuses on underwater 3D reconstruction rather than SR, its degradation-aware self-supervised strategy highlights the importance of adaptive modeling when handling complex real-world degradations. This perspective aligns with the motivation of our degradation-adaptive framework.

### 2.3. SR with Multiple or Unknown Degradations

Real-world degradations are typically more complex than artificially designed ones. The degradation process from HR to LR images can be formulated as follows:

(1) $$I_{LR}=\left(I_{HR}⊗k\right){↓}_{S}+n,\;$$

where *k* represents a blur kernel; ${↓}_{S}$ denotes downsampling; and *n* denotes additive noise [14,32]. Several methods have attempted to model multiple degradations. For example, SRMD [8] incorporates degradation parameters as additional inputs. UDVD [39] employs dynamic convolution to handle cross-image and spatial variations. ZSSR [9] trains image-specific networks during inference, while USRNet [10] adopts a half-quadratic splitting algorithm to alternately solve data and prior sub-problems.

More recent studies have further explored real-world and degradation-aware super-resolution under complex degradation conditions. For example, SeeSR [40] introduced a semantics-aware framework for real-world image super-resolution by combining degradation-aware prompting with generative priors, demonstrating the growing importance of semantic guidance in heavily degraded scenarios. In a broader restoration setting, recent all-in-one restoration methods [41,42] have also shown that unified models can handle multiple degradation types and levels, highlighting the value of adaptive guidance for blind restoration tasks. Nevertheless, degradation estimation remains challenging in blind SR. Estimation errors may be amplified during reconstruction, leading to artifacts such as over-sharpening or over-smoothing [14]. Existing methods often lack sufficient adaptability to complex and unknown degradation patterns observed in historical imagery.

In summary, although significant progress has been achieved in supervised and unsupervised SR methods, most existing approaches rely on synthetic degradation assumptions and limited degradation modeling. They lack flexible adaptation mechanisms for complex, naturally occurring degradations and are typically trained without real historical LR data. These technological and methodological gaps motivate the development of a degradation-adaptive framework tailored for historical image super-resolution.

## 3. Methodology

In this section, we present the unsupervised degradation learning for SISR, which effectively adapts to realistic noise and blur patterns in historical images and generates HR images with finer details and textures. We refer to our framework as Degradation Adaptive CycleGAN for SR (DA-CycleGAN). For clarity, the main mathematical symbols used in this section are summarized in Table 1.

### 3.1. Overview of DA-CycleGAN

**Figure 1:**
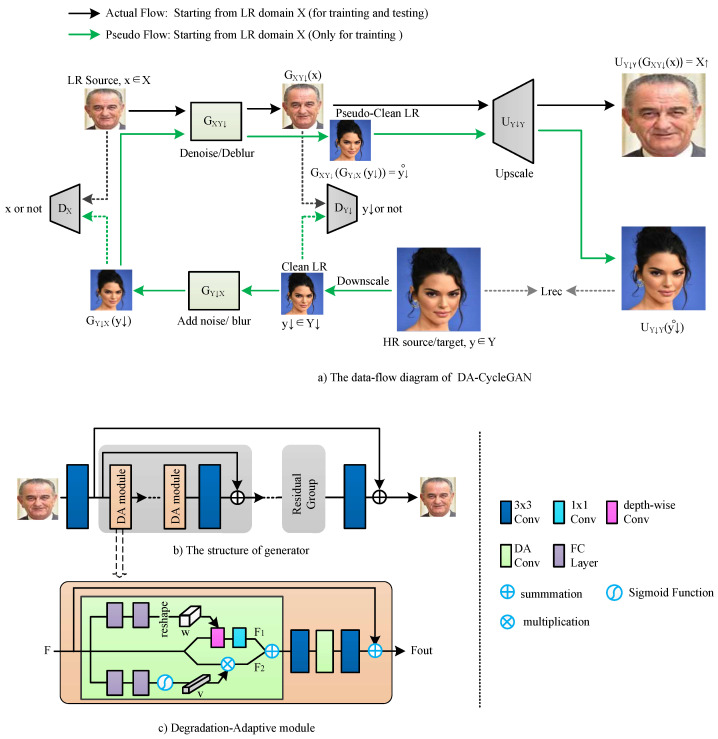
The structure of DA-CycleGAN. (**a**) Data-flow diagram of DA-CycleGAN. (**b**) Generator architecture. (**c**) Degradation-adaptive module structure. In (**a**), solid arrows indicate the main data flow, green arrows denote pseudo flow used during training, and dotted lines represent supervision signals and loss computation pathways.

The proposed DA-CycleGAN network consists of two main parts shown in Figure 1a, namely an unpaired kernel/noise correction network and a pseudo-paired SR network. The correction network is a CycleGAN [43]-based unpaired LR ↔ clean LR translation used to denoise/deblur the LR image. The SR network is a paired mapping from clean LR to HR images, where the clean LR images are generated by downsampling the HR source images. At the training stage, the correction network also produces pseudo-clean LR images. Specifically, the forward generator $G_{Y_{↓}X}$ first maps the clean LR images in domain $Y_{↓}$ to the true LR domain *X*. Then, the inverse generator $G_{XY_{↓}}$ pulls them back to the clean LR domain. The SR network takes the corrected, clean LR images and learns to recover the original HR images from the pseudo-clean LR images in a paired manner.

The DA-CycleGAN network aims to learn a mapping $F_{XY}$ from LR source domain X to HR target domain Y using unpaired training samples *x* (∈X) and *y* (∈Y).

The mapping $F_{XY}$ is decomposed into two mappings, $G_{XY_{↓}}$ and $U_{Y_{↓}Y}$, where $G_{XY_{↓}}$ denotes a denoise/deblur mapping from *X* to $Y_{↓}$, and $U_{Y_{↓}Y}$ represents an upsampling mapping from $Y_{↓}$ to *Y*. The bicubic downsampling operation $Y\to Y_{↓}$, which generates low-resolution (LR) images from high-resolution (HR) images, produces what we refer to as “clean LR”, denoted by $y_{↓}\in Y_{↓}$.

#### 3.1.1. Domain Transfer in LR

We adopt a Pseudo CycleGAN [28]-based model for domain transfer in the LR space. Two generators, $G_{XY_{↓}}$ and $G_{Y_{↓}X}$, are trained simultaneously to learn a pair of opposite mappings with cycle consistency, $G_{XY_{↓}}\left(G_{Y_{↓}X}\left(y_{↓}\right)\right)\approx y_{↓}$.

The discriminators $D_{X}$ and $D_{Y_{↓}}$ are trained to distinguish between translated images and real source samples $y_{↓}$ and *x*, respectively.

#### 3.1.2. Mapping from LR to HR

The upsampling mapping $U_{Y_{↓}Y}$ is trained to reconstruct HR image *y* from the pseudo-clean LR image $G_{XY_{↓}}$($G_{Y_{↓}X}$$\left(y_{↓}\right)$). Hence, the pixel-wise loss functions can be adopted to train upscalor $U_{Y_{↓}Y}$. We denote the operation result of the two inverse mappings $G_{XY_{↓}}$($G_{Y_{↓}X}$$\left(y_{↓}\right)$) as $\overset{˚}{y_{↓}}$.

### 3.2. DA-CycleGAN Network Architecture

To extract various degradations from historical images and restore details of the SR images, we propose to use the degradation-adaptive (DA) module with powerful flexible adaptation in the generative network.

#### 3.2.1. Generators

The generator networks $G_{XY_{↓}}$ and $G_{YX_{↓}}$ are built upon the RCAN architecture [21]. RCAN is a very deep super-resolution (SR) network that employs a residual-in-residual (RIR) structure composed of multiple residual groups with long skip connections, which effectively alleviates redundant low-frequency information during feature extraction in very deep convolutional networks. Figure 1b illustrates the architecture of the proposed generator. The DA module is adopted as the basic building block, and each residual group contains five DA modules. The generator consists of three main components: an initial convolutional block, five residual groups, and a final convolutional reconstruction module.

#### 3.2.2. DA Module

The proposed DA module structure is illustrated in Figure 1c. Based on the observation in this work [44] that filters trained for different restoration levels share similar visual patterns while exhibiting varying statistics (e.g., mean and variance), modulating filter statistics enables continuous control of restoration strength, preventing overly sharp or excessively smooth outputs. Inspired by this, we propose a DA convolutional layer that adaptively modulates the kernel of a depth-wise convolution based on degradation information extracted from the previous feature map.

Specifically, the degradation feature *F* is first fed into two fully connected (FC) layers in the top branch and reshaped to form a convolutional kernel $w\in R^{C\times 1\times 3\times 3}$. Subsequently, the degradation feature *F* is processed using a 3×3 depth-wise convolution with kernel *w*, followed by a 1×1 convolution to produce $F_{1}$.

Furthermore, motivated by the interactive image restoration work CResMD [45], where controllable residual connections allow users to adjust the restoration strength across multiple degradations, our DA convolutional layer learns channel-wise modulation coefficients from degradation features. Specifically, the degradation features are passed through two additional FC layers in the bottom branch, followed by a sigmoid activation to generate channel-wise modulation coefficients *v*. The channel components of *F* are then rescaled by *v* to produce $F_{2}$. Finally, $F_{2}$ is fused with $F_{1}$ and forwarded to subsequent layers to generate the output feature $F_{\mathrm{out}}$.

The proposed degradation-adaptive (DA) module is designed to address complex and unknown degradation patterns in historical images. Unlike conventional super-resolution methods that rely on predefined degradation models or synthetic degradation processes, the DA module adaptively learns degradation-aware representations directly from real-world LR inputs. By dynamically modulating convolutional kernels and channel responses based on degradation features, the proposed module enables the network to adjust its restoration behavior across varying degradation conditions. This adaptive mechanism enables the proposed DA-CycleGAN framework to better handle diverse degradation patterns commonly observed in historical imagery and to improve reconstruction quality compared with existing unpaired SR frameworks.

#### 3.2.3. Discriminators

LR discriminators $D_{X}$ and $D_{Y_{↓}}$ share the same architecture and consist of five convolutional layers. The first four convolutional layers are followed by LeakyReLU activations without Batch Normalization (BN).

### 3.3. Loss Functions

#### 3.3.1. Adversarial Loss

We impose an adversarial loss [46] on generators $G_{XY_{↓}}$ and $G_{Y_{↓}X}$ to generate samples with the goal of fooling their discriminators $D_{Y↓}$ and $D_{X}$, respectively. Take $G_{Y_{↓}X}$ and $D_{Y↓}$ as an example, the adversarial loss can be expressed as follows:

(2) $$\begin{array}{cc} L_{adv}\left(G_{XY_{↓}},D_{Y_{↓}},X,Y_{↓}\right) & =E_{y_{↓}∼P_{Y_{↓}}}\left[\log D_{Y_{↓}}\left(y_{↓}\right)\right]\\ \; & \;+E_{x∼P_{X}}\left[\log\left(1-D_{Y_{↓}}\left(G_{XY_{↓}}\left(x\right)\right)\right)\right]. \end{array}$$

where $P_{X}$ ($P_{Y↓}$) denotes the probability distribution of the domain X$\left(Y_{↓}\right)$. The forward mapping $G_{XY_{↓}}$ and the discriminator $D_{Y↓}$ implement a two-player minimax game to optimize each other, $min_{G_{XY_{↓}}}max_{D_{Y↓}}L_{adv}\left(G_{XY_{↓}},D_{Y↓},X,Y_{↓}\right)$. Similarly, the inverse generator $G_{Y_{↓}X}$ and the discriminator $D_{X}$ are optimized by the GAN loss, $min_{G_{Y_{↓}X}}max_{D_{X}}L_{adv}\left(G_{Y_{↓}X},D_{X},Y_{↓},X\right)$.

#### 3.3.2. Cycle Consistency Loss

We utilize general cycle-consistency loss in a CycleGAN to perform unpaired image-to-image translation (i.e., X → Y → X and Y → X → Y). Considering that our framework needs to handle multiple degradations, that is, various noise types or distributions in the LR source domain X, will be generated. Hence, we impose the cycle consistency constraint for only one side. Cycle consistency loss is expressed as

(3) $$\begin{array}{c} L_{cyc}\left(G_{Y_{↓}X},G_{XY_{↓}}\right)=∥G_{XY_{↓}}\circ G_{Y_{↓}X}\left(y_{↓}\right)-y_{↓}{∥}_{1}. \end{array}$$

With the one-side cycle consistency constraint, $G_{Y_{↓}}$X can perform a one-to-many mapping. Consequently, our framework can deal with various noise types/distributions in the LR source domain X.

#### 3.3.3. Identity Mapping Loss

Identity mapping loss was introduced in the original CycleGAN acting as an effective stabilizer used to preserve the color of the input paintings [43]. In this paper, we impose an identity-mapping loss on $G_{XY_{↓}}$ to avoid altering the color tone of the input image.

(4) $$\begin{array}{c} L_{idt}\left(G_{XY_{↓}}\right)=∥G_{XY_{↓}}\left(y_{↓}\right)-y_{↓}{∥}_{1}. \end{array}$$

#### 3.3.4. Geometry Consistency Loss

Geometry-consistency loss, as a reconstruction loss, first introduced in geometry-consistent generative adversarial network (GcGAN) [47], helps preserve the geometry of a scene for unsupervised domain mapping. We impose the Geometry-consistency loss on $G_{XY_{↓}}$ that allows the flip or rotation will not bring semantic distortions when mapping to target domain:

(5) $$\begin{array}{c} L_{geo}\left(G_{XY_{↓}}\right)={∥G_{XY_{↓}}-\sum_{i=1}^{8}T_{i}^{-1}\left(G_{XY_{↓}}\left(T_{i}\left(x\right)\right)\right)/8∥}_{1}, \end{array}$$

where ${\left\{T_{i}\right\}}_{i=1}^{8}$ represents eight different patterns of flip and rotation of input images.

#### 3.3.5. Full Objective

By combining adversarial constraint with cycle consistency loss, identity mapping loss and geometry-consistency loss, a remarkable unsupervised domain mapping can be targeted. Our full objective for the two generators and its corresponding discriminators is as follows:

(6) $$\begin{array}{cc} L_{trans} & =L_{adv}\left(G_{XY_{↓}},D_{Y↓},X,Y_{↓}\right)\\ \; & +L_{adv}\left(G_{Y_{↓}X},D_{X},Y_{↓},X\right)\\ \; & +\lambda_{cyc}L_{cyc}\left(G_{Y_{↓}X},G_{XY_{↓}}\right)\\ \; & +\lambda_{idt}L_{idt}\left(G_{XY_{↓}}\right)\\ \; & +\lambda_{geo}L_{geo}\left(G_{XY_{↓}}\right), \end{array}$$

where hyperparameters $\lambda_{cyc}$, $\lambda_{idt}$, and $\lambda_{geo}$ represent the contributions of each objective. To reconstruct an HR image from a pseudo-clean LR image generated by the correction network, we use $U_{Y_{↑}Y}$ as an amplifier in the SR network to perform upscaling. The SR network is also updated during the training of the correction network by using L1 loss:

(7) $$\begin{array}{c} L_{rec}={∥U_{Y_{↓}Y}\left({\overset{˚}{y}}_{↓}\right)-y∥}_{1}. \end{array}$$

## 4. Experiments

In this section, we first describe the datasets and implementation details used in our experiments (Section 4.1). We then evaluate the effectiveness of the proposed DA-CycleGAN on the collected historical face image dataset through both qualitative and quantitative comparisons (Section 4.2). To further assess the generalization capability of our method, we conduct additional experiments on two standard benchmark datasets, Set14 (Section 4.2.2) and DIV2K (Section 4.2.3). Finally, we present subjective evaluation results using Mean Opinion Score (MOS) testing and provide a comprehensive analysis of the experimental findings (Section 4.3).

### 4.1. Datasets and Implementation Details

#### 4.1.1. LR Face Image Dataset

**Figure 2:**
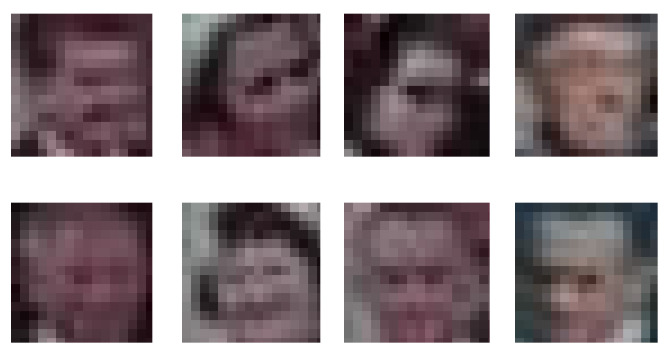
LR samples of the training dataset.

We collected the LR image dataset by cropping faces from a large set of historical video sequences and further processing them with bicubic downsampling. A total of 10,000 LR face images with resolution 16×16 were used as the training set. The 16×16 resolution was selected because faces extracted from historical videos are often extremely small and heavily degraded. This setting allows the model to learn super-resolution under very limited visual information. Figure 2 shows samples from the LR image dataset. The collected LR dataset was divided into training, validation, and testing subsets. Specifically, 8000 images were used for training, 1000 images for validation, and the remaining 1000 images for testing.

The historical video dataset was collected from the United States Marine Corps Film Repository at the Moving Image Research Collections (MIRC), University of South Carolina. The online collection is available at https://digital.library.sc.edu/collections/united-states-marine-corps-films, accessed on 15 January 2023. As discussed in Section 1, due to the limited quality of historical video capture devices, the collected historical images contain multiple types of degradation (e.g., blur, noise, and compression artifacts). Consequently, the generated low-resolution (LR) images inherit diverse degradation characteristics from the original historical high-resolution (HR) real-world images, resulting in richer and more natural degradation patterns, as illustrated in Figure 1.

#### 4.1.2. HR Face Image Dataset

We used the HR face image dataset provided by Bulat et al. [25]. The dataset contains 182,866 HR face images of size 64×64, collected from VGGFace2 [48], AFLW [49], Celeb-A [50], and LS3D-W [51]. All HR face images were cropped using the $S^{3}FD$ face detector [52].

#### 4.1.3. Training Settings and Hyperparameters

Let x∈X denote a real low-resolution (LR) image sampled from the historical source domain *X*, and let y↓∈Y↓ denote a clean LR image obtained by bicubic downsampling from the high-resolution (HR) domain *Y*. For experiments involving real-world degradations, it has been observed that using *x* instead of y↓ as the argument of the identity mapping loss yields better SR performance [28]. Accordingly, the identity mapping loss is defined as:

(8) $$L_{idt}\left(G_{XY_{↓}}\right)={∥G_{XY_{↓}}\left(x\right)-x∥}_{1}.$$

To ensure a fair comparison, we followed the hyperparameter settings of the baseline Pseudo-CycleGAN [28]. The hyperparameters were set to $\lambda_{cyc}=1$, $\lambda_{idt}=2$, and $\lambda_{geo}=1$. The network was trained using 16×16 LR images and 64×64 HR images with a super-resolution scaling factor of ×4. The model was implemented using the PyTorch 2.0.1 deep learning framework. During training, the Adam optimizer was used with a learning rate of $2\times 10^{-4}$ and momentum parameters $\beta_{1}=0.5$ and $\beta_{2}=0.999$. The batch size was set to 16. Training was conducted for 200 epochs. To improve generalization ability, standard data augmentation techniques, including random horizontal flipping and rotation, were applied to the training images. All experiments were performed on a workstation equipped with an NVIDIA A100 GPU.

### 4.2. Effectiveness of the Proposed DA-CycleGAN

#### 4.2.1. Performance Comparison on Historical Image Dataset

**Figure 3:**
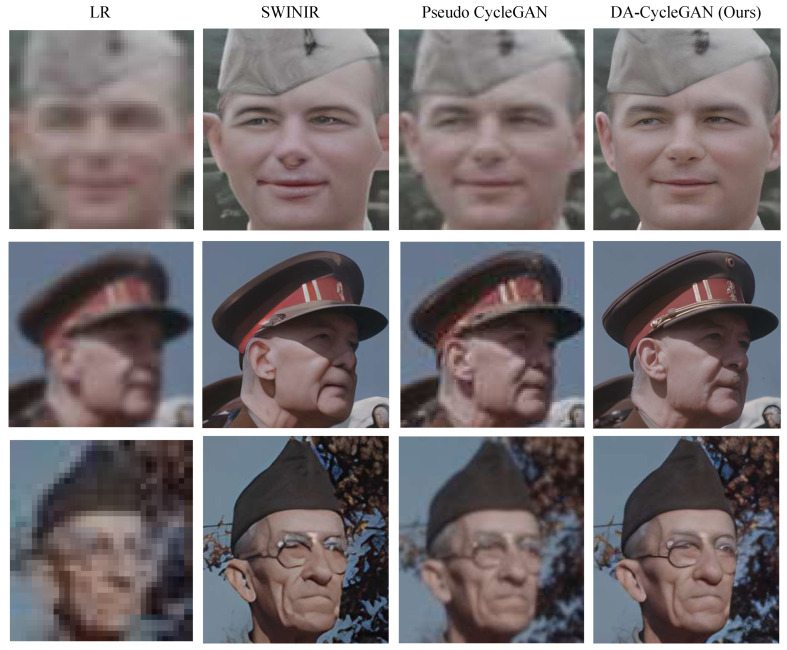
Sample performance comparisons with a state-of-the-art Pseudo CycleGAN [28] on the historical face-image dataset.

This experiment evaluates the SR performance of the proposed DA-CycleGAN on our collected historical image dataset, which contains 10,000 face images collected from the historical video sets. We numerically and visually compared our method with the state-of-the-art CycleGAN-based unpaired pseudo SR method proposed by Maeda [28]. Figure 3 presents example images for the visual performance comparison. It can be seen that our DA-CycleGAN achieves better visual quality than Pseudo CycleGAN [28].

Moreover, we numerically compared our methods with four related state-of-the-art CNN-based or GAN-based methods, including SRGAN [22], SRResNet [22], EDSR [34], RCAN [21]. Given that our historical LR images lack corresponding HR reference (ground-truth) images, traditional full-reference image quality metrics, such as Peak Signal-to-Noise Ratio (PSNR) and Structural Similarity Index (SSIM), are not applicable in this case. Therefore, we adopt a no-reference image quality metric, Neural Side-By-Side [53], to evaluate the restored image quality. Neural Side-By-Side is a pretrained CNN-based model used to measure no-reference image quality by predicting the probability that an image is more preferable than its counterpart. Table 2 shows numerical comparison results on the historical face images dataset. These numerical results indicate that our method produces perceptually better results than Pseudo CycleGAN [28].

#### 4.2.2. Performance Comparison on Set14

**Figure 4:**
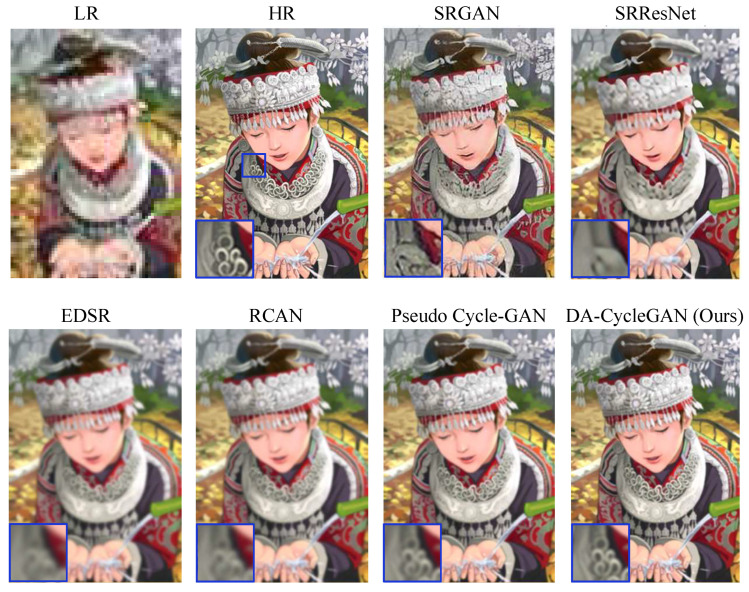
Sample performance comparisons with state-of-the-art SR methods on the Set14 dataset.

To further evaluate the SR performance of the proposed DA-CycleGAN model, experiments were conducted on the widely used Set14 dataset [5]. We compared our method with several state-of-the-art SR approaches on this benchmark (Figure 4). The quantitative results are reported in Table 3 in terms of Peak Signal-to-Noise Ratio (PSNR) and Structural Similarity Index Measure (SSIM).

PSNR is defined as [54]:

(9) $$\mathrm{PSNR}=10\log_{10}\left(\frac{MAX_{I}^{2}}{\mathrm{MSE}}\right),$$

where $MAX_{I}$ denotes the maximum possible pixel value of the image, and MSE represents the mean squared error between the reconstructed image *I* and the ground-truth image $I_{GT}$:

(10) $$\mathrm{MSE}=\frac{1}{N}\sum_{i=1}^{N}{\left(I_{i}-I_{GT,i}\right)}^{2}.$$

PSNR measures pixel-wise reconstruction fidelity, with higher values indicating better reconstruction quality.

SSIM is defined as [55]:

(11) $$\mathrm{SSIM}\left(I,I_{GT}\right)=\frac{\left(2\mu_{I}\mu_{GT}+C_{1}\right)\left(2\sigma_{I,GT}+C_{2}\right)}{\left(\mu_{I}^{2}+\mu_{GT}^{2}+C_{1}\right)\left(\sigma_{I}^{2}+\sigma_{GT}^{2}+C_{2}\right)},$$

where μ and σ denote the mean and variance of the images, respectively, and $\sigma_{I,GT}$ represents the covariance between *I* and $I_{GT}$. $C_{1}$ and $C_{2}$ are constants to stabilize the division. SSIM evaluates perceptual image quality by considering luminance, contrast, and structural similarity [55].

As shown in Table 3, the proposed DA-CycleGAN achieves competitive or superior performance compared with existing SR methods, demonstrating its effectiveness in both reconstruction accuracy and structural preservation.

#### 4.2.3. Performance Comparison on DIV2K

We further evaluate performance on the standard DIV2K benchmark dataset [7], which contains 800 high-quality 2K-resolution training images with diverse content. The realistic-wild LR sets simulate real-world “wild” low-resolution images using ×4 downscaling with additional noise perturbations. The degradation operations remain consistent within each image but vary across different images. Training data are augmented using random flipping and rotation. All compared models are retrained on this dataset for fair evaluation. Performance is evaluated on the DIV2K validation set using PSNR and SSIM metrics. Table 4 reports the quantitative results.

From Table 4, the proposed DA-CycleGAN achieves the best performance among all compared methods. Specifically, DA-CycleGAN improves the PSNR to 23.90 dB and SSIM to 0.8563. Compared with the strongest baseline Pseudo-CycleGAN, the proposed method achieves a PSNR improvement of 0.19 dB and an SSIM improvement of 0.0078. Compared with RCAN, DA-CycleGAN improves PSNR by 0.54 dB and SSIM by 0.0198. These improvements demonstrate that the proposed degradation-adaptive framework is more effective in handling complex degradation patterns in the realistic-wild DIV2K dataset.

#### 4.2.4. Mean Opinion Score (MOS) Testing

**Figure 5:**
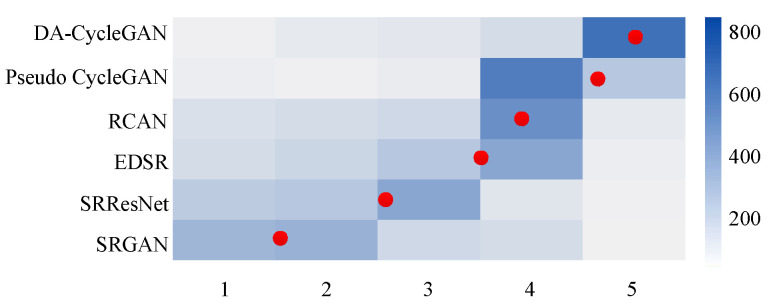
Color-coded distribution of MOS scores on the historical face image dataset. For each SR model, 800 samples (200 images × 4 raters) were rated. Mean shown as red marker, where the bins are centered around value i. [4× upscaling].

To make a fair comparison with existing SR models in terms of the perceptual quality of super-resolved images, we conducted Mean Opinion Score (MOS) testing. MOS is the result of human evaluation of reconstructed images, and the evaluation scores reflect only image quality and do not relate to their content [22]. For MOS testing, we first prepared a subset of LR images: 200 images randomly selected from the historical image dataset, 14 images from Set14, and 186 images randomly selected from the DIV-2k dataset. Then, we employed six different SR models to super-resolve the LR images and obtained six corresponding super-resolved images. Lastly, we asked 4 raters to rate the reconstructed images for perceptual quality on a five-point scale, where 1 is bad and 5 is excellent. For each method 1600 samples (400 images × 4 raters) were assessed. We adopt the average score of MOS test statistics. We found that our MOS test has good reliability as there is no noticeably difference between the ratings of the same image. The experimental results of the MOS test are presented in Table 5 and Figure 5.

#### 4.2.5. Computational Complexity Analysis

In addition to reconstruction performance, we evaluate the computational efficiency of the proposed method. Specifically, we compare the model size (number of parameters), computational complexity (FLOPs), and inference runtime with several representative SR models. Inference time was measured as the average processing time per image. The computational complexity comparison is summarized in Table 6. All methods were evaluated under the same hardware configuration, and the runtime denotes the average inference time per image.

### 4.3. Experiment Results Analysis

Across the historical face dataset, Set14, and DIV2K, DA-CycleGAN consistently achieves strong performance compared with five representative SR baselines, demonstrating its ability to generalize across both synthetic benchmarks and real historical degradations.

On the historical dataset, where ground-truth HR images are unavailable, the proposed method achieves the highest perceptual scores (Neural Side-by-Side and MOS). This indicates that DA-CycleGAN produces visually more convincing reconstructions under complex real-world degradations. Compared with Pseudo-CycleGAN, which serves as the baseline, the additional performance gain suggests that explicitly modeling degradation adaptability plays a critical role in restoring historical imagery.

On benchmark datasets (Set14 and DIV2K), DA-CycleGAN achieves competitive or superior PSNR and SSIM values. The improvement in PSNR reflects enhanced pixel-level reconstruction fidelity, while the gain in SSIM indicates better preservation of structural information. Notably, the performance remains stable across datasets with different degradation characteristics, suggesting improved robustness rather than dataset-specific overfitting.

CNN-based methods such as EDSR and RCAN rely on fixed convolutional kernels learned under predefined degradation assumptions. Although effective for bicubic or synthetic degradations, their performance may deteriorate when confronted with unknown or mixed degradation patterns. GAN-based methods such as SRGAN and SRResNet enhance perceptual sharpness through adversarial learning; however, they may introduce hallucinated textures or instability when the degradation distribution differs from the training conditions.

Pseudo-CycleGAN mitigates the absence of paired data through pseudo-supervision; however, it lacks explicit mechanisms to adapt to varying degradation statistics. In contrast, the proposed degradation-adaptive (DA) module dynamically modulates convolutional kernels and channel responses based on extracted degradation features. This adaptive mechanism enables the network to better handle heterogeneous blur, noise, and compression artifacts commonly observed in historical footage.

Overall, the consistent quantitative improvements and superior perceptual quality demonstrate that incorporating degradation-aware modulation enhances robustness and structural recovery under complex real-world conditions.

## 5. Conclusions

Recent research on single-image super-resolution (SISR) enhancement has progressed with the development of deep learning. However, SISR faces challenges in historical image applications due to the complexity of degradation effects and the lack of paired natural-world training data. In this work, we first presented an efficient unpaired SR model with a degradation-adaptive (DA) block that provides powerful, flexible adaptation to the various complex degradations observed in historical images. Furthermore, to train our model on historical image degradation, we collected an LR historical face images dataset from a large set of historical video sequences, which exhibit real, comprehensive degradation from the physical world. It is demonstrated that our proposed DA-CycleGAN can recover HR images that are visually more convincing than those in previous work. The presented network’s effectiveness makes it well suited as a super-resolver for historical images.

## Figures and Tables

**Table 1:** Summary of mathematical notations.

Symbol	Description
*X*	Real low-resolution (LR) image domain
*Y*	High-resolution (HR) image domain
$Y_{↓}$	Clean LR domain obtained by downsampling HR images
*x*	Sample drawn from LR domain *X*
*y*	Sample drawn from HR domain *Y*
$y_{↓}$	Clean LR image generated from HR image
${\tilde{y}}_{↓}$	Pseudo-clean LR image after correction
$G_{XY_{↓}}$	Backward generator: maps real LR images (*X*) to clean LR domain ($Y_{↓}$)
$G_{Y_{↓}X}$	Forward generator: maps clean LR images ($Y_{↓}$) to real LR domain (*X*)
*U*	Upsampling network: maps $Y_{↓}\to Y$
$D_{X}$	Discriminator associated with LR domain *X*
$D_{Y_{↓}}$	Discriminator associated with clean LR domain $Y_{↓}$
*F*	Degradation feature extracted in DA module
$F_{1},F_{2}$	Intermediate feature maps in DA module
$F_{\mathrm{out}}$	Output feature of DA module
*w*	Dynamically generated depth-wise convolution kernel
*v*	Channel-wise modulation coefficients
$L_{adv}$	Adversarial loss
$L_{cyc}$	Cycle consistency loss
$L_{idt}$	Identity mapping loss
$L_{geo}$	Geometry consistency loss
$L_{rec}$	Reconstruction loss for SR network
$\lambda_{cyc},\lambda_{idt},\lambda_{geo}$	Weighting coefficients of loss terms

**Table 2:** Performance comparison on the historical face-image dataset. Bold text indicates our method.

Method	Human Preference Score
SRGAN [22]	0.06
SRResNet [22]	0.08
EDSR [34]	0.11
RCAN [21]	0.16
Pseudo-CycleGAN [28]	0.28
**DA-CycleGAN (Ours)**	**0.31**

**Table 3:** Performance comparison on Set14. Bold text indicates our method.

Method	PSNR	SSIM
SRGAN [22]	26.02	0.7397
SRResNet [22]	28.49	0.8184
EDSR [34]	28.80	0.7876
RCAN [21]	28.87	0.7889
Pseudo CycleGAN [28]	28.96	0.7913
**DA-CycleGAN (Ours)**	**29.02**	**0.7919**

**Table 4:** Performance comparison on DIV2K. Bold text indicates our method.

Method	PSNR	SSIM
SRGAN [22]	22.66	0.8025
SRResNet [22]	23.10	0.8251
EDSR [34]	23.14	0.8280
RCAN [21]	23.36	0.8365
Pseudo CycleGAN [28]	23.71	0.8485
**DA-CycleGAN (Ours)**	**23.90**	**0.8563**

**Table 5:** Performance comparison evaluated by MOS. Bold text indicates our method.

Method	MOS
SRGAN [22]	2.08
SRResNet [22]	2.39
EDSR [34]	3.07
RCAN [21]	3.35
Pseudo CycleGAN [28]	4.12
**DA-CycleGAN (Ours)**	**4.55**

**Table 6:** Computational complexity comparison with representative SR models.

Method	Parameters (M)	FLOPs (G)	Runtime (ms)
SRGAN [22]	1.5	0.25	8
EDSR [34]	43.1	150.2	35
RCAN [21]	15.6	90.5	28
Pseudo-CycleGAN [28]	11.2	35.4	22
DA-CycleGAN (Ours)	13.0	38.7	24

## Data Availability

The data presented in this study are not publicly available due to historical data restrictions.
